# Challenges affecting the implementation of the Policy on Integration of Mental Health Care into primary healthcare in KwaZulu-Natal province

**DOI:** 10.4102/curationis.v42i1.1847

**Published:** 2019-08-21

**Authors:** Esther N. Hlongwa, Maureen N. Sibiya

**Affiliations:** 1 KwaZulu-Natal College of Nursing, Pietermaritzburg, South Africa; 2 Faculty of Health Sciences, Durban University of Technology, Durban, South Africa

**Keywords:** primary healthcare, mental healthcare, health-related policies, integration of care, comprehensive care integration, service integration

## Abstract

**Background:**

Since the publication of the White Paper for the Transformation of the Health System in South Africa in 1997, which included Policy on Integration of Mental Health Care into primary health care (PHC), there has been an emphasis on the promotion of health as well as the re-engineering of PHC to include the integration of mental health care into PHC. Although South Africa has made significant advances at the level of health-related policy development and legislation in trying to bring the country in line with international trends, there have been challenges with regard to implementation of policies, including that of integration of mental health care into PHC.

**Objectives:**

The aim of this study was to determine the challenges affecting the implementation of the Policy on Integration of Mental Health Care into PHC in KwaZulu-Natal (KZN) province of South Africa and to seek possible solutions.

**Method:**

A qualitative exploratory descriptive design was used to determine the challenges affecting the implementation of the Policy on Integration of Mental Health Care into PHC in KZN. The sample consisted of 42 participants of whom 4 were PHC managers, 6 were operational managers and 22 were professional nurses who were directly involved in implementing the policy at the operational level.

**Results:**

The challenges identified included lack of training in mental healthcare services for staff working in PHC, unavailability of mental health policies, inadequate resources, poor communication between management and staff, lack of skills among PHC nurses in identifying signs of mental illness and misdiagnosis of patients.

**Conclusion:**

Considering the challenges pertaining to PHC nurses’ abilities and skills to implement the Policy on Integration of Mental Health Care into PHC, PHC-trained nurses should engage in lifelong learning and be encouraged to develop their knowledge, skills and competence throughout their professional lives.

## Introduction

Despite the efforts of bringing South African health-related policy development and legislation in line with international trends, there have been many challenges with regard to implementation of policies, including the integration of mental healthcare into primary healthcare (PHC) (Burns 2008:1; Dube & Uys 2015:2; Kigozi & Ssebunnya 2009:37). The Policy on Integration of Mental Health Care into PHC in South Africa was published together with the White Paper for the Transformation of the Health System in South Africa in 1997 (Republic of South Africa 1997). In 2006, the National Department of Health released guidelines for the management of mental disorders (Department of Health 2006:1). In 2013, the National Department of Health in South Africa developed the National Mental Health Policy Framework and Strategic Plan 2013–2020 to guide the implementation of policy framework (Department of Health 2013:1). Many facilities in KwaZulu-Natal (KZN) province have attempted to implement the Policy on Integration of Mental Health Care into PHC, but there are reports of challenges that are facing PHC nurses (Awenva et al. 2012:184; Burns 2008:1; Dube & Uys 2015:2; Kigozi & Ssebunnya 2009:37). This study investigated the challenges that are negatively affecting the implementation of the Policy on Integration of Mental Health Care into PHC.

## Background of the study

Prior to 1994, the healthcare system including mental healthcare in South Africa was fragmented. It was segregated by race or ethnic group and the best resourced facilities were located in urban areas, while rural areas were under-resourced (African National Congress 1994:1). Mental healthcare was characterised by violations of human rights (Burns 2008:45). In 1973, the *Mental Health Care Act* No. 18 of 1973 was promulgated and this act reinforced isolation of the mentally ill patients from general healthcare.

Psychiatric services were stand-alone services and not integrated into PHC (Burns 2008:46). After 1994, changes in the healthcare system as well as mental healthcare took place, and South Africa put more emphasis on comprehensive care. In keeping with the Constitution of South Africa (Republic of South Africa 1996), the White Paper for the Transformation of Health Systems in South Africa was published together with the Policy on Integration of Mental Health Care into PHC in 1997, which made provision for a new health system based on PHC principles (Republic of South Africa 1997:1). The new health system emphasised prevention and promotion instead of being curative and hospital orientated.

In line with international trends, the *Mental Health Care Act* No. 17 of 2002 was promulgated in South Africa based on a number of principles, the fundamental one being that mental healthcare should be integrated into PHC and should be located close to residential areas (Department of Health 2002:1). The Policy on Integration of Mental Health Care into PHC also stipulates that mental healthcare patients should receive treatment at clinics in their residential areas and mental healthcare services must be integrated into PHC (Republic of South Africa 1997:1). This demands that PHC-trained professional nurses, working at the clinic, should be able to assess all patients, identify patients with mental illness and provide the necessary treatment. Although South Africa has made significant advances at the level of health-related policy development and legislation in trying to bring the country in line with international trends, there have been challenges with regard to implementation of policies, including that of integration of mental healthcare into PHC.

## Problem statement

Since the publication of the White Paper for the Transformation of the Health System in South Africa in 1997, which included Policy on Integration of Mental Health Care into PHC, there has been an emphasis on the promotion of health as well as the re-engineering of PHC to include the integration of mental healthcare into PHC. Primary healthcare engineering in South Africa is a national strategy which was launched in 2010 by the National Minister of Health, aimed at improving PHC and promoting healthcare which is more practical, integrated and based on the needs of the population (Department of Health 2010:1). In a PHC clinic, nurses are responsible for the management, administration and counselling of patients (Department of Health 2010:2). Since the publication of the Policy on Integration of Mental Health Care into PHC, there have been many challenges (Burns 2008:1; Dube & Uys 2015:2; Kigozi & Ssebunnya 2009:37). Challenges reported included insufficient support and information to guide PHC-trained nurses on the implementation of the policy, and PHC-trained nurses lacking the necessary skills to provide quality mental healthcare (Awenva et al. 2012:184; Petersen et al. 2011:4; Williams et al. 2008:211). This prompted a need to identify, investigate and address these challenges that are affecting the integration of mental healthcare, and facilitate implementation of the integration of mental healthcare into PHC in KZN.

## Aim of the study

The aim of the study was to determine the challenges affecting the implementation of the Policy on Integration of Mental Health Care into PHC in KZN.

## Objective of the study

The objectives of the study were to:

- Explore challenges that hinder the implementation of the policy on integration of mental healthcare into PHC as perceived by PHC nurses in KZN province.
- Explore possible solutions from the perspective of PHC nurses to effectively implement the Policy on Integration of Mental Health Care into PHC in KZN province.

## Research questions

The study was guided by the following questions:

- What are the challenges that affect the implementation of the policy on the integration of mental healthcare into PHC?
- What are the possible solutions for ineffective implementation of the Policy on Integration of Mental Health Care into PHC?

## Significance of the study

From the literature review, it was evident that there is poor integration of mental healthcare into PHC. Various studies have indicated that PHC services are ineffective in managing mental healthcare users, despite all the guidelines that have been developed (Burns 2008:1; Dube & Uys 2015:2). Primary healthcare–trained nurses are at the forefront of the provision of PHC at the clinics. No previous study has been conducted to determine challenges affecting the implementation of the Policy on Integration of Mental Health Care into PHC by PHC nurses in KZN. The findings of this study will provide insight into the challenges experienced by the PHC nurses in implementing the Policy on Integration of Mental Health Care into PHC and offer possible solutions.

## Operational definitions

For the purpose of this study, it is necessary to define the concepts that were used throughout the article to ensure that the readers and the researchers share the same meaning attached to specific concepts.

### Mental healthcare

Mental healthcare is a human clinical science based on a variety of theoretical frameworks with emphasis on the psychosocial and bio-physical sciences. It constitutes knowledge regarding promotion of mental health and primary, secondary and tertiary prevention of mental illness (Townsend 2015:908). For the purpose of this study, mental healthcare refers to the care given to mentally ill patients by nurses working in PHC clinics.

### Primary healthcare

Primary healthcare is usually the first point of contact people have with the healthcare system that provides essential health services accessible to individuals, families and community through their full participation and at a cost that the community and country can afford to maintain at every stage of their development. This includes a broad spectrum of services from promotion of health to prevention of illnesses and management of chronic diseases (World Health Organization 2018:1). For the purposes of the study, PHC refers to the comprehensive care given to all patients at the nearest clinic.

### Mental health

Townsend (2015:907) defines mental health as a successful adaptation to stressors from internal or external environments evidenced by thoughts, feelings and behaviours that are age appropriate and congruent with local and cultural norms. For the purposes of this study, mental health means to be successful in working, loving, resolving conflicts and adapting well to the environment.

### Service integration

Service integration is defined as bringing together of services and activities that share common goals (Sibiya & Gwele 2009:31).

### Policy implementation

Policy implementation is defined as a complex change process whereby government decisions are transformed into programmes, procedures, regulations and practices that are aimed at the betterment of society and the community at large (Khan & Khandaker 2016:1).

## Research methodology

### Design

A qualitative, exploratory, descriptive and contextual design was used to explore the challenges affecting the implementation of the Policy for the Integration of Mental Health Care into PHC in KZN. The design was chosen to achieve the aim of the study, which was to provide an in-depth description of the phenomenon under study. The design was followed for this study considering that very little is known about the challenges that affect the implementation of the policy on the integration of mental healthcare into PHC.

### Study setting

The study took place in health districts in KZN which were purposefully selected, according to their geographical location, because the boundaries of these health districts coincide with the district and metropolitan municipal boundaries. The districts selected included: central (eThekwini), midlands (uMgungundlovu), north (iLembe) and south (uGu). eThekwini and uMgungundlovu health districts are situated in urban areas and iLembe and uGu health districts are situated in semi-rural areas. It is often assumed that health facilities that are located in urban areas are better resourced than facilities located in rural areas. All four districts had implemented the policy on the integration of mental healthcare into PHC at their clinics.

### Population and sampling process

A three-stage sampling approach was applied: the first stage was selecting health districts in KZN which included central (eThekwini), midlands (uMgungundlovu), north (iLembe) and south (uGu). All four districts had implemented the Policy on the Integration of Mental Health Care into PHC at their clinics.

The second stage of sampling was to select clinics located within the districts of eThekwini, uGu, uMgungundlovu and iLembe. Purposive sampling was used to select four clinics. Two clinics in the rural areas and two in the urban areas were selected, and the criteria for selection were as follows: the clinics should provide comprehensive care and have implemented the Policy on Integration of Mental Health Care into PHC. The researcher purposefully selected one central and accessible clinic per district that provides comprehensive care, and which had implemented the Policy on Integration of Mental Health Care. The clinics that were purposefully selected were KwaDabeka Clinic in eThekwini, Gamalakhe Clinic in the uGu district, Mbalenhle Clinic in the uMgungundlovu district and Ndwedwe Clinic in the iLembe district.

The third stage of sampling was to select participants who were willing to take part in the study. Purposeful sampling was used to select participants. Primary healthcare managers, operational managers as well as professional nurses at the selected clinics participated in this study. Purposeful sampling was the ideal sampling technique to select participants as the cadres of healthcare workers selected are involved in the implementation of the Policy on Integration of Mental Health Care into PHC at the selected clinics.

### Data collection process

After the researcher had obtained ethical clearance from the university and the KZN Department of Health, data were collected from the four PHC clinics in each district. The sample consisted of 42 participants of whom 4 were PHC managers, 6 were operational managers and 32 were professional nurses who were directly involved in implementing the policy at the operational level. The researcher interviewed the PHC managers and the operational managers, and a total of five focus group interviews were conducted with the professional nurses, four with six participants and one with eight participants. The researcher continued with interviews and focus group discussions until data saturation was reached. Data saturation occurs when sampling provides no new information and the data collected become redundant (Grove, Burns & Gray 2013:371). In this study, data saturation was reached when the researcher interviewed the third PHC manager, the fifth operational manager and conducted the seventh focus group interview. This is when the researcher realised that the participants were repeatedly giving the same information.

A guide was used to facilitate the one-on-one interviews and focus group discussions. Data obtained during the one-on-one interviews and focus group discussions were voice-recorded using field notes as a backup. Data collection and analysis occurred concurrently, allowing the researcher to constantly compare similarities and variations to determine common categories and subcategories.

### Data analysis

The recorded one-on-one interviews and focus group interviews were transcribed and analysed according to Tesch’s method of open coding (Creswell 2012:191). Data from the participants were coded together and checked by the supervisor who has experience in qualitative research. The main themes and sub-themes were identified. Conclusions and recommendations were then drawn from the data.

### Trustworthiness

To ensure trustworthiness of the study, Lincoln and Guba’s model was used (Polit & Beck 2012:584). The strategies used in the study to ensure trustworthiness were credibility, transferability, dependability and conformability. Credibility was achieved in this study by double checking of audio-recording, field notes and member checking. The researcher gave feedback to the participants regarding the themes that were emerging from the data in order to obtain reactions and to explore if these interpretations were a good representation of the participants’ reality. To give the reader a full understanding of the study, a full description of the research process was provided. This is supported by Brink, Van der Walt and Van Rensburg (2012:57) who state that before processing of data, the researcher must examine them for completeness and accuracy. Transferability was ensured by ensuring that there was a description of the research setting, the participants of the study and a full description of results.

To ensure dependability of the study, the research process was carefully documented through the use of field notes, voice recordings and the description of the role of the researcher. Different methods of collecting data were used which included one-on-one interviews and focus group interviews. Conformability was achieved by ensuring that the results were confirmable, procedures were correctly documented and data checked during data collection and analysis.

### Ethical considerations

Ethical approval for conducting this study was obtained from the ethics committee (Ethical clearance reference number: REC 46/16). Permission to conduct the study was requested from the District Manager and the KZN Department of Health. All participants in the study were given a letter of information and consent form to consent to participate in the study. Participation was voluntary and the participants were told that they can withdraw from the study at any time without any penalty. The researcher requested permission to voice record the interviews. The participants were informed that they would not receive monetary benefits for participating in the study. Extra precautions were taken to safeguard the participants with regard to anonymity and confidentiality. Their names were not mentioned and recorded during discussions. They were advised of the confidentiality and anonymity of the discussion and responses.

## Findings

Themes that emerged from the data analysis are discussed below.

### Lack of skills in managing mental healthcare users

The study revealed that PHC nurses who were managing patients at the clinic were not adequately competent to offer all the services that are offered in clinics where a one-stop-shop approach was used. This is supported by Lund et al. (2012:403) who reported that some mental health patients are treated by registered nurses who do not have qualifications in Psychiatric Nursing Science and that this practice may lead to mental health patients being misdiagnosed, leading to relapse of mental illness.

The following are some challenges expressed by interviewees:

‘Not everyone who works in the clinic has Psychiatric Nursing Science qualification or has participated in the in-service training. A number of PHC nurses do not have a qualification in psychiatric nursing.’ (PHC manager, Interview, P4)

‘Currently, there is no trainer to assist staff in mental health training. Not every professional nurse is good in mental health care.’ (Operational manager, Interview, P1)

‘When it comes to human resources, there are not enough resources to deal with all these things, staff that is willing to implement the policy. Nurses must be trained, at the moment if one person has gone for training, that person needs to come back and cascade the information at PHC level.’ (Operational manager, Interview, P2)

Primary healthcare–trained nurses, as evident from the above excerpts, felt neglected and concerned that they are not competent in mental healthcare as expected.

### Unavailability of mental health policies for staff at the clinic

Unavailability of mental health policies for staff at the clinic was cited as a limiting factor in implementing the Policy on Integration of Mental Health Care into PHC, resulting in a lack of knowledge of the policy. At some of the clinics that the researcher visited, participants expressed concern that they had not seen the Policy on Integration of Mental Health Care into PHC and the *Mental Health Act*, although they had heard of these. The results of this study are consistent with the study conducted by Dube and Uys (2015:7) whose findings revealed that mental health guidelines, including the *Mental Health Care Act* (No. 17 of 2002), were not available in four of the study sites in South Africa. One can assume that participants expected managers to make policies accessible and they also needed to be educated on the policy and the act.

Participants responded as follows:

‘We don’t have access to policies. We have not seen the policy. I think not being aware of the policy, lack of access to information and policies is not good. I think managers should make sure that their staff have access to information.’ (Professional nurse, FGD, P32)

‘We don’t have access to access to intranet. No one talks to us about policies for mental health. We have HIV policies instead.’ (FGD, P7)

‘We have no access to policies and Acts and most of us have not seen the policy and we don’t understand contents of these policies you are talking about.’ (Professional nurse, FGD, P30)

### Inadequate resources

Inadequate resources emerged as a factor hindering the implementation of integration of mental healthcare into PHC. Participants from rural clinics reported shortage of space, consulting rooms and medication to treat mental illness. This was different from clinics in urban areas where there was enough space and consulting rooms. Lack of space results in lack of privacy which interferes with the ability of staff to counsel mental health patients.

All clinics cited shortage of staff, especially psychiatric nurses, specialist psychiatrists and advanced psychiatric nurses, as the main factor hindering the implementation of integration of mental healthcare into PHC. In some clinics, a psychiatrist only comes in once a week and a general practitioner sees all patients including mental healthcare users.

‘We have shortage of staff, only two professional nurses in Out Patients Department, if one goes for lunch or sick, only one is left alone. Shortage of psychiatrists and no advanced psychiatric nurses.’ (Operational manager, Interview, P3)

‘There is inadequate space and consulting rooms suitable for counselling. We have inadequate staff and space in this clinic, no medication to give to mentally ill patients., (Professional nurse, FGD, P10)

### Poor communication between management and staff

Poor communication between management and staff emerged as a factor hindering the implementation of the integration of mental healthcare into PHC. The participants said that there was a communication gap between managers and staff at grass roots level. In contradiction, managers expressed satisfaction with how managers and staff communicate. This is supported by Temane, Poggenpoel and Myburg (2014:7) who conducted a study with advanced psychiatric nurse practitioners on their ideas and needs for supervision in private practice in South Africa and found that supervisors lacked communication skills; they suggested that there is a need for supervisors to possess facilitative communication skills.

‘Not being aware of the policy and lack of access to information. Management is not communicating with staff, not informing staff about new policies.’ (Professional nurse, FGD, P4)

‘Managers do not communicate with us staff members. No policies are communicated to us. It’s the first time somebody has spoken to us.’ (Professional nurse, FGD, P17)

‘I feel managers should communicate with us, even if once a month. At this clinic, we only work. They only communicate with us when they give us work.’ (Professional nurse, FGD, P6)

### Lack of skills of managing mental healthcare users among primary healthcare nurses

Another factor hindering the implementation of the integration of mental healthcare into PHC was PHC nurses’ lack of skills to manage mental health patients. It was found that PHC nurses’ lack of skills in identifying signs of mental illness, misdiagnosing and giving wrong treatment or failure to treat mental health patients led to relapse of poor support, and lack of skills in identifying patients with signs of mental illness has been cited by Burns (2008:1) as an important factor undermining the implementation of integration of mental healthcare into PHC.

‘Not every professional nurse knows how to nurse mental health care users. A number of professional nurses do not have skills and ability to diagnose and treat mentally ill patient.’ (PHC manager, Interview, P1)

‘Lack of skills in identifying mental health care users, misdiagnosing them and this leads to treating them with medication that will not relieve the symptoms.’ (Professional nurse, FGD, P5)

### Possible solutions that will allow integration of mental healthcare into primary healthcare to take place effectively

**FIGURE 1:**
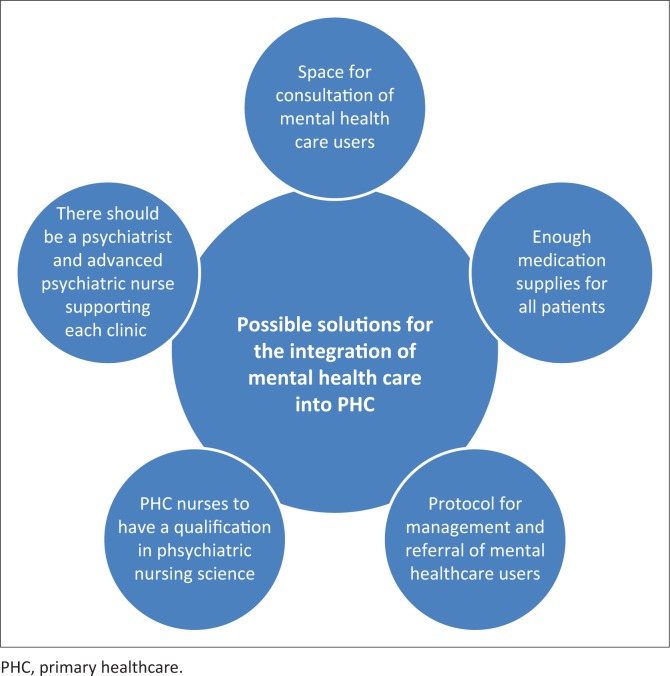
Possible solutions for the integration of mental healthcare into primary healthcare.

The participants cited conditions that need to be in place for the implementation of the Policy on Integration of Mental Health Care into PHC to be effective. These include adequate and appropriate space or consulting rooms for patients at the clinic, adequate supply of medication for all patients, protocol for the management and referral of mental healthcare users, qualification in Psychiatric Nursing Science and PHC for all nurses working at the clinic, and there should be more psychiatrists and advanced psychiatric nurses. Figure 1 summarises possible solutions for the integration of mental healthcare.

### Adequate space or consulting rooms for patients at the clinic

Participants recommended that each PHC clinic must provide adequate space and consulting rooms for counselling and interviewing clients, which will allow effective integration of mental healthcare into PHC. Privacy allows confidentiality of information between the mental health patient and health worker to be achieved. Participants reported as follows:

‘Environment should be conducive and safe for counselling and management of patients’ space must be able to accommodate difficult patients and allows for management of agitated and aggressive patients in the clinic.’ (Professional nurse, FGD, P11)

‘We need more consulting rooms and bigger spaces especially because other members of the multi-disciplinary teams visit the clinic: we really need more space to accommodate everybody.’ (Operational manager, Interview, P6)

### Adequate medication for all patients

Participants suggested that in each PHC clinic, there should be adequate medication supplies for all patients. Adequate medication supplies allow PHC nurses to give quality treatment to mental health patients and prevent relapse and re-admission to mental institutions as noted in the excerpts below:

‘There should be enough treatment available for all patients at the clinic. Enough medication needs to be stored in every consulting room even for night duty or weekends.’ (PHC manager, Interview, P1)

‘Shortage of medication and at times some of the drugs used by mental health care users is not in stock. Medication is important for managing mental health care users all the time.’ (Operational manager, Interview, P1)

### Protocol for management and referral of mental healthcare users

Protocol for management and referral of mental healthcare users is an important condition that needs to be in place before integration of mental healthcare can take place. In each clinic, there must be step-by-step procedures formulated by management for the management and referral of mental healthcare users and this must be communicated to all staff at the clinic. Engagement of community members and other stakeholders in the community, including police, on how to manage mental healthcare users, should be arranged so that all stakeholders support the policy.

‘There should be step-by-step protocols on how to manage and refer mental health care users in each clinic, and there must be enough resources to implement the protocol.’ (Professional nurse, FGD, P25)

‘If the mental health care user cannot be treated, then the mental health care user needs to be referred and there must be a proper protocol in place to determine when the patient must be referred.’ (Operational manager, Interview, P5)

‘There should be protocols that are communicated throughout the department by management.’ (Professional nurse, FGD, P14)

### Qualification in Psychiatric Nursing Science and primary healthcare for all nurses working at the clinic

Qualification in Psychiatric Nursing Science for all nurses working at the PHC clinic was identified as an important condition for integration of mental healthcare into PHC to be effective. Participants recommended that PHC nurses should be trained to identify signs of mental illness as noted in the quotes below:

‘PHC nurses must be trained in both Psychiatric Nursing Science and Primary Health Care to be able to understand and manage all patients including mental health care users.’ (PHC manager, Interview, P2)

‘Every professional nurse at the clinic must also have knowledge of mental illness and must be able to recognise signs of mental illness.’ (Operational manager, Interview, P6)

‘It is important to train nurses in psychiatric nursing so that they don’t misdiagnose mental illness.’ (Operational manager, Interview, P1)

### The availability of advanced psychiatric nurses and psychiatrists at each clinic

Participants recommended that in each PHC clinic, there must be psychiatrists and advanced psychiatric nurses to provide support and mentoring of PHC staff who have basic qualifications in Psychiatric Nursing Science and PHC. According to Marais and Petersen (2015:15), there are insufficient specialist personnel to support professional nurses who have basic qualifications and those who do not have qualification in psychiatric nursing.

‘I think that PHC nurses need support and mentoring from the psychiatrists or advanced psychiatric nurses. At the moment there is only one PHC nurse with advanced psychiatry.’ (PHC manager, Interview, P1)

‘The psychiatrist only comes only comes once a week and this is not enough. I think we are having problems to implement the policy because of lack of support from Psychiatrists.’ (Professional nurse, FGD, P23)

‘I think PHC nurses need specialist support for effective integration of mental health care into PHC.’ (Operational manager, Interview, P3)

## Discussion

Integration of mental healthcare remains a critical issue in the healthcare system in KZN province despite many efforts by the National and Provincial Departments of Health. The study revealed that the phenomenon of integration of mental healthcare into PHC was seen by PHC-trained nurses as provision of comprehensive care that is either given through a supermarket approach or a one-stop-shop approach, depending on the availability of PHC nurses with a qualification in psychiatric nursing. The study also revealed that the majority of PHC nurses who were managing patients at the clinic were not adequately trained to offer all the services that were offered at a one-stop-shop. This is consistent with the findings of Lund et al. (2012:403) who reported that some mental healthcare users are seen by registered nurses who do not have a qualification in Psychiatric Nursing Science and further argue that this practice may lead to mental healthcare users being misdiagnosed, leading to a relapse of mental illness.

This study reported challenges affecting the Policy on Integration of Mental Health Care into PHC as perceived by PHC-trained nurses in selected health districts in KZN province. Challenges identified included lack of training in mental healthcare services for staff working at PHC, unavailability of mental healthcare policies for staff at the clinic, inadequate resources, poor communication between management and staff as well as lack of skills among PHC nurses in identifying signs of mental illness. The findings of this study are consistent with the study conducted by Dube and Uys (2015:8), which explored practices used by PHC nurses in the management of mental healthcare users at selected PHC clinics in uThungulu District in KZN. Their study revealed that there was no evidence of thorough assessment of mental healthcare users and argued that this could be attributed to PHC nurses lacking knowledge and skills in managing mental healthcare users. Xaba, Peu and Phiri (2012:11) and Marais and Petersen (2015:6) highlighted the lack of training and support of staff in providing comprehensive care and recommended that healthcare workers should be appropriately trained and supported to render services that are otherwise beyond their level of training.

Inadequate resources emerged as hindering the implementation of integration of mental healthcare into PHC. Participants from rural clinics reported shortage of space, consulting rooms and medication to treat mental illness. This was different from participants from clinics in urban areas who reported having enough space and consulting rooms to maintain privacy. Lack of space results in lack of privacy, which interferes with the ability of staff to counsel mental healthcare users.

All clinics cited shortage of staff (especially psychiatric nurses, specialist psychiatrists and advanced psychiatric nurses) as the main factor hindering the implementation of integration of mental healthcare into PHC. In some clinics, a psychiatrist comes once a week only and a general practitioner sees all patients including mental health patients. Shihundla, Lebese and Maputle (2016:7) maintain that the Department of Health should have strategies to retain their staff to prevent shortages of staff and increased workload. This is supported by Igumbor et al. (2016:7) who state that innovative strategies are necessary to combat the shortage of healthcare professionals and improve the healthcare system in South Africa.

Lack of communication between management and staff was a factor hindering the implementation of the integration of mental healthcare into PHC. The participants were of the view that there was a communication gap between managers and staff at the grass roots level. The results of this study are consistent with the study conducted by Mmamma, Mothiba and Nancy (2015:4), which revealed that professional nurses experienced a lack of support from top management during the execution of nursing duties at the hospital. These authors further suggest that top management should support staff members rather than intimidating them. Similarly, Sojane, Klopper and Coetzee (2016:1) found that nurses felt unsupported and undervalued and suggested that nurse managers can improve job satisfaction by giving praise and recognition at work. Lack of communication about the policy on integration of mental healthcare into PHC at district level has been reported by Marais and Petersen (2015:14), who suggested that this can be improved by including strategic planners in development plans that have the capacity to translate policies into plans at provincial and district levels.

Primary healthcare nurses’ lack of skills in identifying signs of mental illness, misdiagnosing and giving wrong treatment, or failure to treat leading to relapse of the mental healthcare users, emerged as a factor hindering the effective integration of mental healthcare into PHC. Currid et al. (2011:21) reported that PHC nurses in London have poor skills in detecting and treating people with mental disorders. Xaba et al. (2012:6) found that in Tshwane (Gauteng), there is a shortage of PHC nurses, psychologists and psychiatrists at clinics and newly qualified nurses are left on their own without support and mentoring. Al-Khatham et al. (2013:204) argued that if mental illness is not detected, it can lead to difficulties in managing it.

Adequate medication supplies for all patients were viewed as an important condition that needs to be in place before integration of mental healthcare can take place. The participants stated that enough medication supplies allow PHC nurses to give treatment to mental healthcare users and prevent relapse and re-admission to mental institutions. Marais and Petersen (2015:10) state that there are major challenges with regard to medicines and technologies related to the supply of psychotropic medicines at district level. In most instances, this is attributed to a breakdown in communication between the hospitals, clinics and pharmacies. These authors further state that lack of medication supplies leads to mental healthcare users defaulting which may lead to relapse.

## Limitations

One of the limitations of this study is that it did not include mental healthcare users and their families as they are the custodians of integration of mental healthcare into PHC. It would be of great interest to see how mental healthcare users perceive and understand integration of mental health into PHC. It would also be informative if general patients who are receiving treatment at PHC clinics together with mental healthcare were included to find out their perceptions of this policy.

Another limitation is that, of the 11 districts in KZN province, this study only looked at two rural districts and two urban districts; therefore, the study results cannot be generalised to other settings.

## Recommendations

Despite the limitations that might impact the generalisability of the results, the following are some recommendations based on the findings of the study.

### Nursing practice

Based on the findings of this study, there is evidence that a lack of communication between management and operation staff exists. Despite the benefits of the Policy on Integration of Mental Health Care into PHC, a lack of communication regarding policies and provisions of the *Mental Health Act* may have a serious effect on the implementation of the policy. Therefore, KZN needs to have protocols, guidelines and communication strategies on how to implement the policy. Managers need to be capacitated with requisite skills to provide leadership in this matter.

Lack of supervision by specialists can also hamper the provision of quality mental healthcare at PHC.

Therefore, it is recommended that there should be more resident psychiatrists and advanced psychiatric nurses who can supervise and mentor PHC nurses in providing quality mental healthcare.

Lack of resources, space, equipment, lack of political will and increased workloads were identified as major factors hindering the implementation of the policy. It is recommended that PHC facilities should receive increased budgets to provide adequate and additional services at PHC clinics.

### Nursing education

The growing population in South Africa together with the increasing burden of disease requires additional services that need to be provided by PHC nurses. This situation often creates increased workloads, where there are shortages of staff, especially trained staff. The training of more nurses with adequate skills is critical. On the basis of the findings of this study, it is recommended that the PHC curriculum should be re-examined so that new PHC graduates understand the integration of mental healthcare into PHC, their roles in this regard and how to function in such an environment.

### Future research

This study has revealed that there are challenges affecting the implementation of the Policy on Integration of Mental Health Care into PHC. The researcher recommends that future research should be conducted on the effect of the implementation of the Policy on Integration of Mental Health Care into PHC on mental healthcare users and their families.

## Conclusion

This study attempted to analyse the challenges that affect the implementation of the Policy on Integration of Mental Health Care into PHC with the aim of suggesting solutions that will enhance the implementation of the policy in KZN, South Africa. Considering the challenges pertaining to PHC nurses’ abilities and skills to implement the Policy on Integration of Mental Health Care into PHC, PHC-trained nurses should engage in lifelong learning and be encouraged to develop their knowledge, skills and competence throughout their professional lives.
